# MicroRNAs in kidney development and disease

**DOI:** 10.1172/jci.insight.158277

**Published:** 2022-05-09

**Authors:** Débora Malta Cerqueira, Maliha Tayeb, Jacqueline Ho

**Affiliations:** 1 Division of Nephrology, Department of Pediatrics, University of Pittsburgh School of Medicine, Pittsburgh, Pennsylvania, USA.; 2 John G. Rangos Sr. Research Center, UPMC Children’s Hospital of Pittsburgh, Pittsburgh, Pennsylvania, USA.

## Abstract

MicroRNAs (miRNAs) belong to a class of endogenous small noncoding RNAs that regulate gene expression at the posttranscriptional level, through both translational repression and mRNA destabilization. They are key regulators of kidney morphogenesis, modulating diverse biological processes in different renal cell lineages. Dysregulation of miRNA expression disrupts early kidney development and has been implicated in the pathogenesis of developmental kidney diseases. In this Review, we summarize current knowledge of miRNA biogenesis and function and discuss in detail the role of miRNAs in kidney morphogenesis and developmental kidney diseases, including congenital anomalies of the kidney and urinary tract and Wilms tumor. We conclude by discussing the utility of miRNAs as potentially novel biomarkers and therapeutic agents.

## Introduction

MicroRNAs (miRNAs) are endogenous small noncoding RNAs that are usually 19–22 nucleotides in length. The human genome contains 1917 annotated hairpin precursors and 2654 mature miRNAs (1), which regulate over 60% of human protein-coding genes (2). MiRNAs regulate gene expression at the posttranscriptional level, through both translational repression and mRNA destabilization (3–5). Since the discovery of the function of the first identified miRNA, which was shown to regulate cell lineage decisions in the nematode *Caenorhabditis elegans*, in 1993 (6, 7), miRNAs have been demonstrated to modulate diverse biological processes, including kidney morphogenesis. Dysregulation of miRNA expression disrupts early kidney development and has been implicated in the pathogenesis of developmental kidney diseases. In this Review, we summarize current knowledge on miRNA biogenesis, function, and targeting. We then focus on the role of miRNAs in kidney morphogenesis and developmental kidney diseases, including congenital anomalies of the kidney and urinary tract (CAKUT) and Wilms tumor. Additional interesting areas of research, including the role of miRNAs in a variety of other kidney diseases, such as acute kidney injury (8–10), polycystic kidney disease (11), and kidney transplant (10), have been extensively addressed in other recent reviews. Finally, we conclude by discussing the utility of miRNAs as potentially novel biomarkers and therapeutic agents.

## MiRNA biogenesis and function

**Figure 1:**
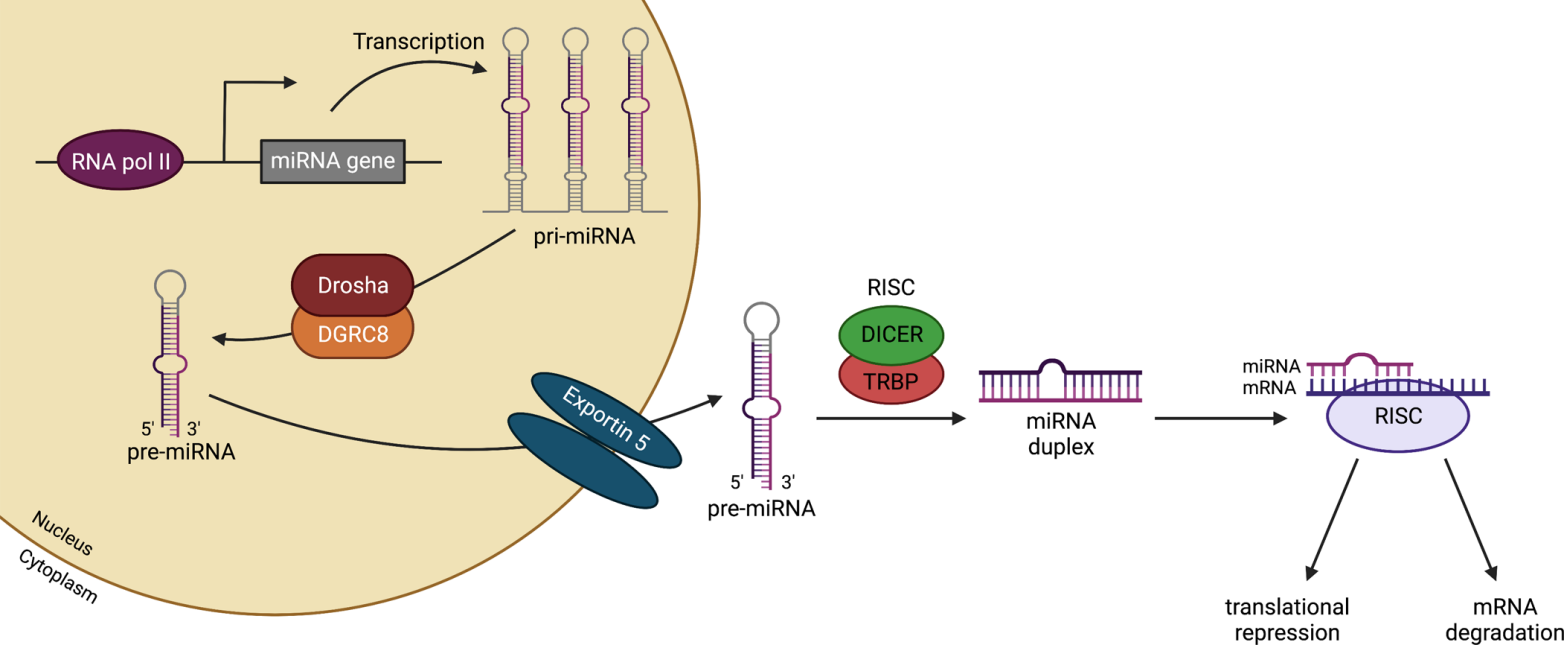
Biogenesis of miRNAs. MiRNA-encoding genes are transcribed by RNA polymerase II into a primary miRNA (pri-miRNA). Next, a complex formed by the RNA-binding protein DGRC8 and the RNase III enzyme Drosha cleaves the pri-miRNA, generating precursor miRNA (pre-miRNA), which is exported into the cytoplasm through exportin 5. Once in the cytoplasm, the Dicer/TRBP complex cleaves the pre-miRNA, releasing mature miRNA. Finally, the mature miRNA is loaded onto the RISC, driving target mRNA recognition through Watson-Crick base pairing, culminating in gene silencing through translational repression or mRNA degradation. DGRC8, DiGeorge syndrome critical region 8; RISC, RNA-induced silencing complex; TRBP, TAR RNA-binding protein. Created with BioRender.com.

Biogenesis of miRNAs begins in the nucleus, where RNA polymerase II transcribes miRNA-encoding genes into capped and polyadenylated hairpin transcripts, named primary miRNAs, or pri-miRNAs (Figure 1) (12, 13). Depending on their genomic location, miRNA-encoding genes can be classified as intragenic (located within the introns of host genes; ref. 14) or intergenic (transcribed independently of a host gene and having their own transcriptional regulatory elements; ref. 15). In addition, some miRNAs exist in clusters and are transcribed as polycistronic transcripts (16).

Following transcription, the pri-miRNA is cleaved by the ribonuclease III–like enzyme DROSHA together with the microprocessor complex subunit DGRC8 into a 70-nucleotide hairpin structure, called a pre-miRNA (17–20). The exportin 5/GTP-binding nuclear protein RAN exports the pre-miRNAs to the cytoplasm (21, 22), where the pre-miRNA undergoes cleavage of its terminal loop by the RNase III DICER and TRBP (or TARBP2) to produce a 22-nucleotide miRNA duplex consisting of guide and passenger strands (miRNA:miRNA*, respectively). In the next step, the miRNA duplex is loaded onto an argonaute (AGO) protein to form the RISC (23). Following strand selection and unwinding, the passenger strand is released and degraded (24), while the guide strand remains in the RISC and drives target mRNA recognition through Watson-Crick base pairing.

Most studies show that the domain at the 5′ end of miRNAs (termed the seed sequence, which extends from nucleotide positions 2 to 7) interacts with a specific region at the 3′ untranslated region (3′UTR) of their target mRNAs to induce translational repression and/or mRNA deadenylation and decay (3–5). However, miRNA binding sites have also been identified in other regions, including the 5′UTR (25, 26), coding sequences (27), and gene promoters (28–30). Although miRNAs are primarily associated with gene repression, posttranscriptional upregulation by miRNAs can also occur under certain circumstances (28, 31–33).

There are several unique features associated with miRNA-mediated gene regulation (34, 35). First, a single miRNA can target and repress hundreds of mRNAs, albeit typically to a relatively mild degree for each individual target. Thus, miRNAs are thought to function to fine-tune gene expression. However, as each mRNA can encompass multiple binding sites for the same or different miRNAs, the resultant combined effect is more potent (36–38). Moreover, multiple components within a given signaling pathway can be modulated by individual miRNAs or miRNA clusters (39, 40). Second, miRNA-mediated repression occurs relatively rapidly, as miRNAs block protein synthesis at the ribosome level (41). Third, miRNAs can be concentrated in specific subcellular compartments to regulate site-specific protein translation (42, 43). Finally, a small subset of miRNAs dominates the total miRNA pool in various cell types, suggesting that these may function as master miRNAs (44). In keeping with this idea, a few of the most abundant miRNAs appear to comprise the majority of posttranscriptional regulation mediated by AGO proteins in many cell types (44, 45). For example, in an immortalized human embryonic kidney cell line (HEK293T), miRNAs that were expressed below 100–1000 reads per million did not demonstrate suppressive activity using a high-throughput miRNA sensor library (45).

Biogenesis of miRNAs is under tight spatial and temporal control to ensure appropriate miRNA expression in response to various cellular signals. Regulation of miRNA biogenesis occurs at multiple levels, including transcription factor binding to enhancers and/or promoters of miRNA genes, DROSHA processing of pri-miRNAs, DICER processing of pre-miRNAs, RNA methylation, editing of miRNA precursors, adenylation, uridylation, RNA decay, and many other mechanisms. For an in-depth review, please refer to Ha and Kim (46). Recently, super-enhancers have also emerged as a new class of regulatory elements controlling miRNA biogenesis by enhancing both transcription and DROSHA/DGCR8-mediated pri-miRNA processing. In combination with a broad H3K4me3 signature, super-enhancer activity shapes tissue-specific miRNA expression pattern and function (47).

## Kidney development

The mammalian kidney (or metanephros) is a vital organ that plays a critical role in excretion of metabolic wastes, regulation of extracellular fluid volume, and maintenance of electrolyte and acid-base homeostasis. Moreover, the kidney produces important hormones, such as erythropoietin, calcitriol, renin, and prostaglandins (48). The functional capacity of the kidney correlates with the number of functioning nephrons that are formed during kidney development prior to birth, also termed nephron endowment. Each human kidney contains on average 1,000,000 nephrons, although this number varies considerably, with estimates ranging from 200,000 to 2,000,000 nephrons (49, 50). With aging, loss of functional nephron reserve occurs over time (51, 52); therefore, low nephron endowment at birth is associated with an increased risk of developing hypertension and chronic kidney disease (CKD) later in life (53–55). Moreover, CAKUT, which lead to decreased nephron endowment and nephron function, are the leading causes of renal failure in children, resulting in significant morbidity and mortality associated with transplant and dialysis (56, 57). Thus, a better understanding of the cellular and molecular mechanisms underlying the establishment of nephron number and normal nephron formation provides insights into novel avenues to predict, prevent, and treat childhood kidney disease.

**Figure 2:**
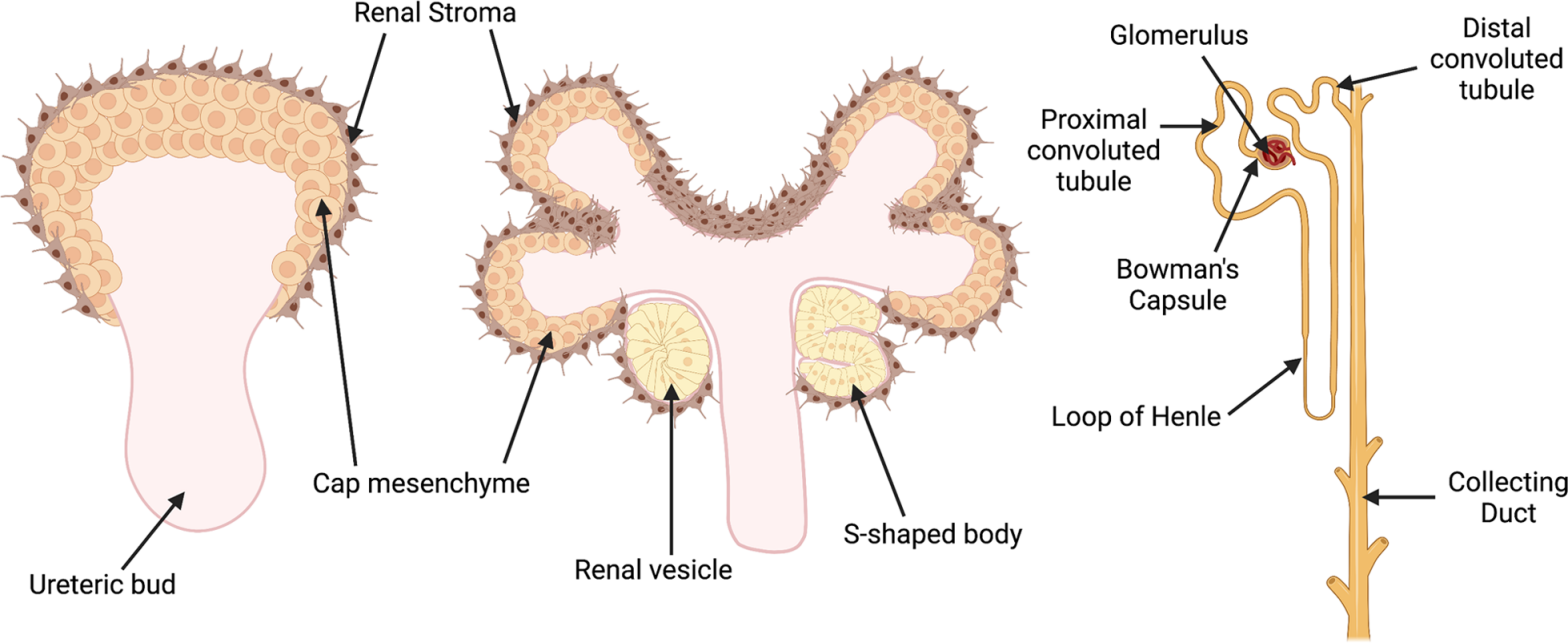
Schematic illustration of the stages of metanephric kidney development. Signals from the ureteric bud trigger condensation of the metanephric mesenchyme to form a cap of nephron progenitors (cap mesenchyme) around the ureteric bud tips. The cap mesenchyme undergoes a mesenchymal-epithelial transition to form renal vesicles, which develop sequentially into comma- and S-shaped bodies. These structures connect to the ureteric bud stalk, which give rises to the collecting duct. Cells in the proximal domain of the S-shaped body differentiate into specialized epithelial cells of the mature renal corpuscle (i.e., podocytes and Bowman’s capsule cells), while cells in the mid- and distal portions differentiate into the tubular segments of nephron (proximal tubules, loops of Henle, and distal tubules). Created with BioRender.com.

Metanephric kidney development starts around embryonic day 10.5 (E10.5) in mice and around the fifth week of gestation in humans (58). In response to inductive signals from the metanephric mesenchyme, the ureteric bud extends from the caudal end of the Wolffian duct and invades into the adjacent mesenchyme (Figure 2). Simultaneously, morphogens emanating from the ureteric bud induce condensation of the metanephric mesenchyme to form the cap mesenchyme (also termed nephron progenitors) around the tips of the ureteric bud. As nephrogenesis progresses, the ureteric bud undergoes successive rounds of branching, elongation, and differentiation to generate the collecting ducts of the kidney. A subpopulation of nephron progenitors undergoes mesenchymal-epithelial transition to form renal vesicles, which after polarization and elongation become comma- and S-shaped body structures. Finally, the distal portion of the S-shaped body fuses with the collecting duct to form a functional nephron (59–61). The S-shaped body undergoes further differentiation to form the mature cell types of the nephron, apart from the collecting duct. *Foxd1^+^* stromal progenitor cells are adjacent to nephron progenitors in the outer cortical or nephrogenic zone of the developing kidney (Figure 2) (62). Signals from the cortical stroma are thought to inhibit nephron progenitor cell expansion and stimulate its differentiation, as ablation of the renal stroma results in impaired nephron progenitor differentiation (63). The *Foxd1^+^* progenitor cells give rise to all stromal cells in the metanephric kidney, including renal cortical and medullary interstitial cells, pericytes, perivascular fibroblasts, mesangial cells, and vascular smooth muscle cells (64, 65). Perturbations in any step of this process can lead to CAKUT, the major cause of childhood CKD (66, 67).

The mature nephron is composed of a glomerulus that acts as the filtration unit and a tubular reabsorption compartment that is subdivided into proximal convoluted tubule, loop of Henle, distal convoluted tubule, and collecting duct (Figure 2). The filtration barrier of the glomerulus (which comprises the fenestrated endothelium, glomerular basement membrane, and foot processes and slit diaphragms of podocytes) allows the filtration of plasma and small solutes, while selectively retaining proteins such as albumin and immunoglobulins in the blood (68, 69). Meanwhile, the tubular reabsorption compartment is responsible for maintenance of water homeostasis, reabsorption of solutes (including sodium, potassium, calcium, phosphorous, magnesium, glucose, and many others), and excretion of acid and other wastes.

## MiRNAs in the developing kidney

In studies in conditional transgenic mice, miRNAs have emerged as critical regulators of kidney morphogenesis in multiple cell lineages. The initial studies evaluating a functional role for miRNAs in kidney development utilized conditional deletion of *Dicer* (70) in different renal lineages. However, *Dicer* is also known to have miRNA-independent roles (71), which has complicated the interpretation of these models. Conditional deletion of *Dicer* in early metanephric mesenchyme or nephron progenitors results in augmented apoptosis of nephron progenitors, elevated levels of the proapoptotic protein Bim, and premature cessation of nephrogenesis (72–75) (Table 1). Interestingly, the loss of Bim expression in *Dicer*-deficient nephron progenitors decreases apoptosis and partially restores nephron formation. Two miRNAs expressed in nephron progenitors, *miR-17* and *miR-106*, were identified as suppressors of BIM expression (76). Together, these findings indicate that miRNAs control the balance between survival and apoptosis in nephron progenitors to ensure the formation of a correct number of nephrons throughout nephrogenesis.

Conditional deletion of *Dicer* in the ureteric bud lineage results in a spectrum of abnormalities that strongly resemble CAKUT, including renal dysplasia and the development of collecting duct cysts (73, 77, 78). Premature termination of branching morphogenesis (in response to decreased expression of *Wnt11* and *c-Ret* from the ureteric bud) is likely the major contributing factor for renal dysplasia (73). The onset of cyst formation occurs at around E15.5 and is associated with defects in primary cilia length, increased apoptotic cell death, and excessive cell proliferation (73). As *Dicer* has important miRNA-independent roles in the cell, conditional deletion of *Dgcr8* has been used to confirm that the phenotypes observed in conditional *Dicer*-knockout models are indeed the result of loss of miRNAs. Animals with *Dgcr8* deletion in the distal nephron and derivatives of the collecting duct develop hydronephrosis and collecting duct cysts (79), a CAKUT-like phenotype that resembles loss of Dicer activity in the ureteric bud lineage.

Studies from two independent groups demonstrated that ablation of *Dicer* from the *Foxd1^+^* renal stroma lineage and its derivatives results in a spectrum of renal anomalies, with consistent findings regarding hypoplastic kidneys, reduced glomerular numbers, abnormal glomerular maturation, and defective vascular patterning (80, 81). Though both groups described largely concordant phenotypes using similar mouse models, two distinct differences were noted. Nakagawa et al. observed a lack of the inner medulla and papilla, as well as a decrease in the nephrogenic zone (80). In contrast, Phua et al. showed an expansion of the nephron progenitor population and preserved renal papilla (81). Nakagawa et al. proposed that these defects are related to disruption of Wnt pathway signaling, resulting in changes in stromal cell migration and proliferation, due to downregulation of the stromal cell miRNAs, *miR-214*, *miR-199a-5p*, and *miR-199a-3p* (80). The study by Phua et al. suggested that changes in apoptotic programs (including augmented expression of *Bim* and p53 effector genes) contribute to the phenotypic defects (81). It is conceivable that genetic background differences and/or the efficiency of Cre-mediated recombination may be responsible for the differences these studies describe. Nevertheless, the described phenotypes are consistent with the known multifaceted roles of the renal stroma in kidney development, and it is likely that the mechanisms underlying these phenotypes are complex given the nature of a *Dicer* deletion. Further studies examining specific miRNAs in various stromal subpopulations are needed to better define the regulatory mechanisms at play.

More recent work has addressed the question of the function of specific miRNAs in both the developing kidney and nephron progenitors. Using a human embryonic stem cell model, Bantounas et al. showed that inhibition of the *miR-199a~214* cluster results in dysmorphic glomeruli, aberrant proximal tubules, decreased WT1 expression, and increased interstitial capillaries in kidney-like organoids (82). Interestingly, global deletion of hypoxia-responsive *miR-210* results in a male-specific nephron deficit (83). For example, conditional deletion of the *miR-17~92* cluster in nephron progenitors and their derivatives in mice impairs progenitor cell proliferation and reduces the number of developing nephrons. As a result, mutant mice develop proteinuria, renal fibrosis, and impaired renal function (84). Dysregulated levels of the *miR-17~92* target gene, *Cftr*, are implicated in defective proliferation of progenitor cells and reduced nephron endowment in this mouse model (85).

Small RNA sequencing (smRNA-Seq) has been increasingly used to profile miRNA expression patterns and for the discovery of novel miRNA species. smRNA-Seq of E15.5 nephrogenic mesenchymal cells identified 162 annotated miRNAs that are differentially expressed in this cell population compared with whole kidney and 49 novel miRNA species (86). Interestingly, levels of *miR-200* family miRNAs were significantly reduced in nephron progenitors. Given that members of the *miR-200* family have been shown to be key regulators of mesenchymal-epithelial transition in the collecting duct (87), we speculate that their expression might be tightly regulated to ensure normal epithelial differentiation of nephron progenitors during kidney development.

## MiRNA function in the mature nephron

In addition to their requirement during kidney development, miRNAs regulate numerous biological processes in the major cell lineages that form the mature nephron (69, 88–91). In keeping with this, segment-specific expression of miRNAs along the nephron has been described, including *miR-143* and *miR-195a* in the glomerulus, *miR-107* and *miR-34a* in the proximal tubule, *miR-193* and *miR-378a* in the thick ascending limb, *miR-874* and *miR-155* in the distal convoluted tubule, and *miR-200c* in the collecting duct (87). Moreover, functional studies in compartments of the mature nephron support distinct roles for miRNAs.

Mice lacking either *Dicer* or *Drosha* in podocytes exhibit marked proteinuria, glomerulosclerosis, and rapid progression to kidney failure, secondary to disruption of the glomerular filtration barrier (90–93). In silico analyses reveal that various upregulated transcripts in mutant glomeruli contain target sequences for *miR-30* family members. As all four *miR-30* family members (*miR-30c-1*, *miR-30b*, *miR-30d*, and *miR-30c-2*) are normally highly expressed in podocytes, these miRNAs may be responsible for the podocyte abnormalities and disruption of the glomerular filtration barrier in mutant mice (91).

Somewhat surprisingly, deletion of *Dicer* from postnatal mammalian proximal tubules does not affect kidney development, histology, or function but does protect against renal ischemia/reperfusion injury. Mutant mice exhibit better kidney function, reduced kidney injury, lower tubular apoptosis, and improved survival compared with their WT littermates (94). This likely reflects the “sum” of the effect of deletion of multiple miRNAs in the proximal tubule, as other work has since demonstrated that the expression of specific miRNAs is protective in renal ischemia/reperfusion injury (e.g., miR-16 and miR-21; refs. 95, 96); whereas others are injurious (e.g., miR-182; ref. 97).

Although miRNAs seem to be dispensable for proximal tubule function, they are essential for distal nephron and collecting duct homeostasis (79, 88, 98). Collecting duct-specific inactivation of *Dicer* and other critical miRNA biogenesis-associated genes (including *Dgcr8*, *Ago1*, *2*, *3*, and *4*) causes renal failure in adult mice because of progressive tubulointerstitial fibrosis and interstitial inflammation (88). This is preceded by a partial epithelial-mesenchymal transition (EMT) of collecting duct cells, and downregulation of *miR-200* family members, which inhibit EMT (88). Likewise, ablation of either *Dicer* or *Dgcr8* from distal nephron and ureteric bud derivatives, respectively, results in renal abnormalities and kidney failure (78, 98), which are ultimately associated with downregulation of *miR-200* family members (98). Increased expression of *miR-200* target gene *Pkd1* in these mutant mice disrupts tubulogenesis and produces cyst-like structures (98). These differences in the requirement for functional miRNAs in proximal tubules and distal nephron/collecting duct might be explained by the segmental distribution of miRNAs along the length of the nephron and collecting duct in WT kidneys (88).

Deletion of *Dicer* in renin-secreting cells in the juxtaglomerular apparatus results in a deficit of juxtaglomerular cells, reduced circulating renin levels with consequent reduction in arterial blood pressure, reduced kidney function, striped pattern of interstitial fibrosis, and vascular abnormalities (89). The reduction in juxtaglomerular cells suggests a requirement for mature miRNAs in the maintenance of their phenotype. Later, *miR-330* and *miR-125b-5p* were identified as potential candidates that either inhibit or promote, respectively, the smooth muscle phenotype of juxtaglomerular cells (99).

Other active areas of research on miRNAs in acute kidney injury (8–10), polycystic kidney disease (11), and kidney transplant (10), have been comprehensively addressed in other recent reviews.

## MiRNAs in pediatric kidney diseases

In this section, we provide an overview of the role of miRNAs in developmental kidney diseases, including CAKUT and Wilms tumor. CAKUT are among the most frequent form of malformations at birth, affecting approximately 3–7 out of 1000 live births (100). Disruption of kidney and lower urinary tract development leads to a wide spectrum of clinical manifestations observed in CAKUT, including kidney anomalies (i.e., renal agenesis, renal hypoplasia and dysplasia, and multicystic dysplastic kidneys), ureteropelvic anomalies (i.e., ureteropelvic junction obstruction), duplex collecting system, and anomalies of the bladder and urethra (101–103). This phenotypic heterogeneity is likely due to complex interactions between genetic, epigenetic, and/or prenatal environmental factors that affect kidney and lower urinary tract development, resulting in CAKUT (101). Most of our current knowledge on CAKUT pathogenesis has arisen from mouse models and syndromic forms of CAKUT. These studies have led to the identification of several CAKUT genes, many of which are implicated in early kidney development, including *PAX2*, *SALL1*, *HNF1B*, *EYA1*, *GATA3*, *RET*, *WNT4*, *GDNF*, *SIX1*, *SIX2*, and others (101, 104, 105). However, single mutations or copy number variants in protein-coding genes do not explain the majority of CAKUT cases (~80%) (101, 106).

As mentioned above, depletion of mature miRNAs from different cell lineages of the developing kidney in mouse models results in renal abnormalities that mimic human CAKUT (73, 74, 78, 79). In addition, germline deletions of *MIR17HG*, which encodes the *miR-17~92* cluster, causes type 2 Feingold syndrome in humans (107). Although a renal phenotype in type 2 Feingold syndrome patients with *MIR17HG* mutations remains undefined, an 18% incidence of CAKUT has been reported in Feingold syndrome cases associated with *MYCN* mutations (108, 109). Together, these observations suggest that mutations in miRNAs expressed during kidney development might cause CAKUT in humans, particularly as many miRNAs are highly conserved between mouse and human.

To test this hypothesis, one study investigated 1248 patients with nonsyndromic CAKUT from 980 families and looked for mutations in 96 stem-loop regions of 73 renal developmental miRNA genes (106). Within this cohort, 31 individuals with 17 different single nucleotide variants affecting 16 different miRNA genes were identified. Among these, two novel variants in miRNAs were found to be potentially pathogenic. *MIR19B1* (a member of the *miR-17~92* cluster) was associated with the presence of right renal agenesis, and *MIR99A* was associated with severe vesicoureteral reflux and kidney ptosis. This surprisingly low number of candidate pathogenic variants is partly due to limitations of this study, as the analysis only accounted for mutations in miRNA genes that were included in the candidate gene approach and did not detect copy number variations and large DNA rearrangements (106).

In an alternative approach, ureter segments from patients with a variety of CAKUT were analyzed for differential transcript expression via microarray, for the presence of bioinformatically predicted miRNA targets, and for mature miRNAs via qPCR (110). Using this multipronged approach, seven miRNAs were identified with potential roles in CAKUT, and among these, *hsa-miR-144* was significantly increased in patients with CAKUT. Gene ontology analysis indicated that predicted *hsa-miR-144* target genes contribute to biological processes involved in CAKUT development, including tube development (22 target genes), urogenital system development (18 target genes), kidney development (14 target genes), and embryonic organ development (18 target genes) (110).

Further studies are needed to define the molecular mechanisms underlying the pathogenic roles of miRNAs in CAKUT. Findings from such studies will be critical in improving the care of patients with CAKUT and preventing their progression to CKD, providing appropriate genetic counseling for patients and their families, and developing novel therapeutic strategies.

## MiRNAs in Wilms tumor

Wilms tumor, or nephroblastoma, is the most common childhood renal cancer, with an incidence of 1 in 10,000 children in North America (111). It is primarily a sporadic disease, although familial forms occur in approximately 1%–2% of cases (112, 113). Wilms tumors arise from aberrant nephrogenesis, where pluripotent embryonic renal precursors fail to differentiate and persist abnormally into postnatal life (111, 114, 115). These tumors histologically resemble developing kidneys with a disrupted morphology (116), and mutations in genes involved in fetal nephrogenesis, including *WT1* (117–119), *CTNNB1* (120), *SIX1*/*2* (121, 122), and *TP53* (123, 124), are associated with approximately 40% of Wilms tumors (125). Recent studies using whole-genome and whole-exome sequencing of Wilms tumors identified novel mutations in cancer risk genes (*REST*, *CHEK2*, *PALB2*) (126, 127), genes encoding proteins that mediate histone modifications during nephrogenesis (*BCOR*, *MAP3K4*) (126), and miRNA-processing genes (121, 122, 128, 129).

**Figure 3:**
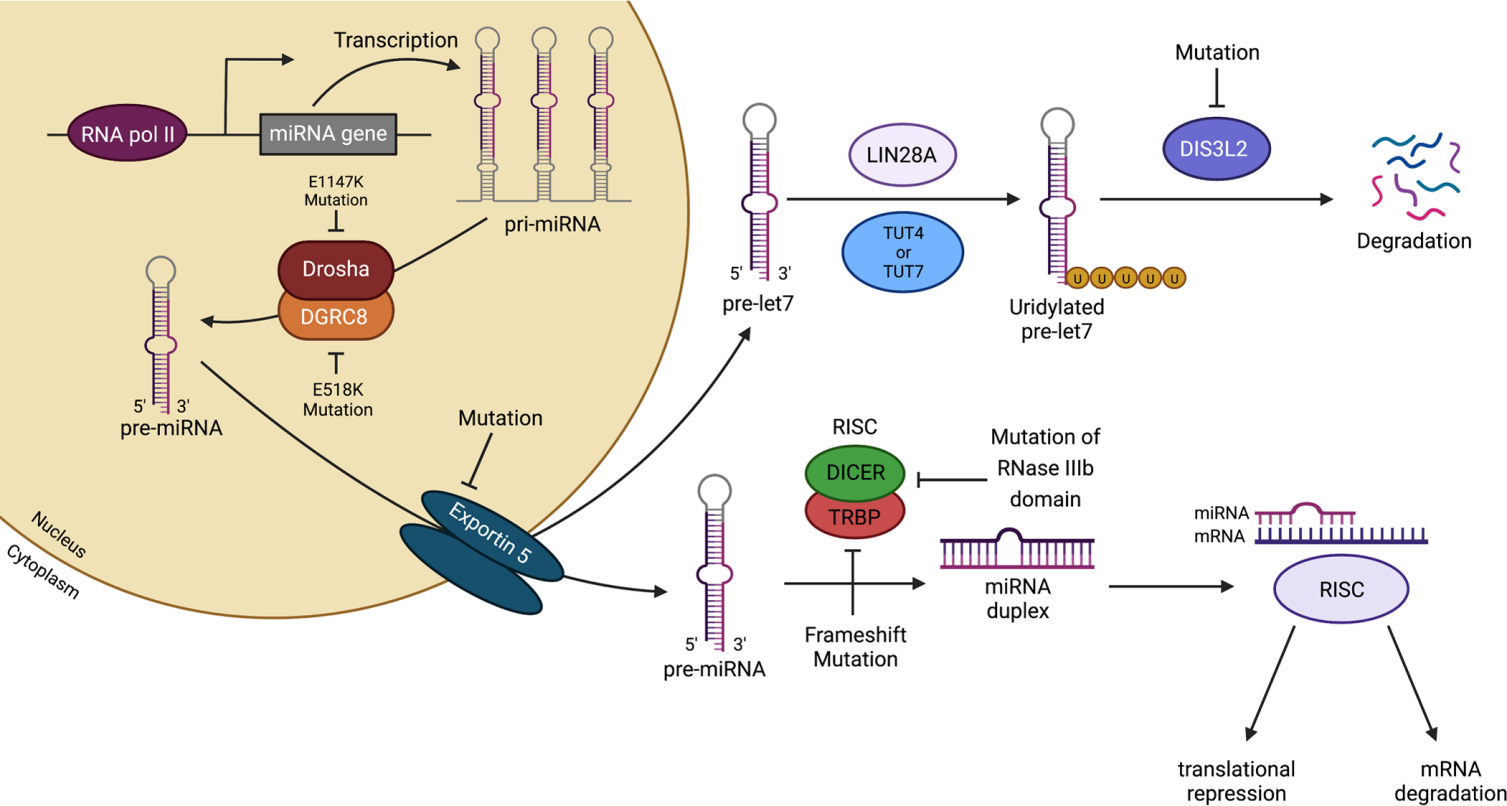
Mutations in miRNA-processing genes result in aberrant miRNA expression and Wilms tumorigenesis. Recurrent mutations in a metal-binding (Mg^2+^) residue of the RNase IIIb domain of *DROSHA* (*E1147K*) or in the double-stranded RNA-binding domain of *DGRC8* (*E518K*) disrupt the cleavage of pri-miRNAs into pre-miRNAs. Mutations in *XPO5* (encodes exportin 5) prevent pre-miRNA export, which culminates in pre-miRNA accumulation in the nucleus. Frameshift mutations in *TARBP2* (encodes TRBP) and mutations affecting the RNase IIIb domain of *DICER1* can disrupt the processing of pre-miRNAs into mature miRNAs. In stem and progenitor cells, members of the *let-7* miRNA family function as tumor suppressors, and their expression is tightly regulated by the RNA-binding protein Lin28. Lin28A binds to the terminal loop of *let-7* precursors and recruits the activity of the terminal uridyl transferases TUT4/7 to produce uridylated *pre-let-7*, which is subsequently degraded by DIS3L2. Overexpression of *LIN28* and mutations in *DISL3L2* have been associated with aberrant mature *let-7* expression and Wilms tumorigenesis. Created with BioRender.com.

Among the miRNA-processing genes, mutations in *DROSHA*, *DGCR8*, *DICER1*, *TARBP2*, and *XPO5* (encodes exportin 5) have been reported in treatment-naive and neoadjuvant chemotherapy-treated Wilms tumors (Figure 3) (121, 122, 128, 129). About 33% of Wilms tumors examined exhibit deleterious mutations in genes of the miRNA-processing pathway (128). A recurrent hotspot mutation (*E1147K*) in a metal-binding (Mg^2+^) residue of the RNase IIIb domain of *DROSHA*, which appears to be unique to Wilms tumor, abolishes the catalytic activity of this domain, resulting in incomplete cleavage of pri-miRNAs and reduced miRNA maturation (128). Somatic hotspot mutations affecting the RNase IIIb domain of DICER1 impair processing of 5p miRNAs (those derived from the 5′-arm of the pre-miRNA hairpin) (129) and are often found as “second hit” mutations that act in tandem with *DICER1* germline mutations to induce Wilms tumorigenesis in DICER1 syndrome (a disorder that increases susceptibility to a variety of tumors) (130–132). It remains unclear why impaired DICER1 function in Wilms tumors results in persistent and aberrant nephrogenesis, unlike loss of *Dicer1* in mouse nephron progenitors, which causes increased apoptosis and a premature cessation of nephrogenesis (72–75). Some potential possibilities include that the gene dosage activity might be crucial in determining cell survival or that mutations in the RNase IIIb domain might affect the specificity of miRNA binding by DICER1.

Mutations in miRNA-processing genes are associated with downregulation of important miRNAs, including members of the *miR-200* (121) and the *let-7* families (Figure 3) (121, 129). *Let-7* miRNAs and the RNA-binding protein Lin28 function in concert to control the timing of cessation of murine nephrogenesis, possibly via regulation of the growth-promoting gene *Igf2* (133). Overexpression of Lin28 during kidney development causes expansion of nephrogenic progenitors, by inhibiting their final wave of differentiation, which culminates in neoplastic transformation that is highly reminiscent of human Wilms tumor (134). Increased DNA copy number of *LIN28B* and DNA copy loss of *let-7a* are seen in 25% and 46% of human Wilms tumor samples, respectively (126). In line with these observations, germline mutations in the human *DISL3L2* gene, which encodes an exoribonuclease responsible for degrading preprocessed forms of *let-7*, cause Perlman syndrome and predisposition to Wilms tumor (135, 136). Perlman syndrome is a congenital overgrowth syndrome characterized by macrosomia, polyhydramnios, facial dysmorphology, renal dysplasia, and nephroblastomatosis (a precursor lesion for Wilms tumor) (137). Among infants with Perlman syndrome who survive past the neonatal period, 64% develop Wilms tumor (138). Interestingly, complete or partial *DISL3L2* deletions were found in about 30% of sporadic Wilms tumors examined (136).

A recent study strengthened the significance of the miRNA regulatory network in the etiology of Wilms tumor. The authors found that pleiomorphic adenoma gene 1 (*PLAG1*) is one of the most consistently upregulated genes in Wilms tumors with mutations in miRNA-processing genes (125). Ectopic expression of *PLAG1* in the developing mouse kidney causes neoplasia, which is accompanied by transactivation of its target gene, the Wilms tumor oncogene *IGF2*. *miR-16* and *miR-34*, which are downregulated in Wilms tumors, were identified as potential regulators of *PLAG1* expression (125). Table 2 summarizes other studies that have reported aberrant expression of specific miRNAs associated with the etiology of Wilms tumor. Interestingly, these miRNAs can function as oncogenes (called oncomiRs) or tumor suppressors in the setting of Wilms tumor development, depending on the nature of their targets.

## MiRNAs as potential biomarkers and therapeutic agents

Apart from their intracellular location, miRNAs are also present in significant amounts in biological fluids, including blood, plasma, urine, breast milk, and saliva (139). These circulating miRNAs are found packaged in microparticles (exosomes, microvesicles, and apoptotic bodies) (140, 141), conjugated with AGO (142) or nucleophosmin 1 proteins (143), or loaded into HDL (144), which make them remarkably stable even under unfavorable conditions, such as boiling, extreme variations in pH, extended storage, and multiple freeze-thaw cycles (145, 146). Thus, miRNA signatures in biological fluids can reflect associations with physiological or disease conditions (147). Together, these features make circulating miRNAs attractive for use as noninvasive biomarkers for disease diagnosis and prognosis.

Circulating miRNAs can be extracted directly from unfractionated biological fluids or from extracellular vesicle preparations using commercially available extraction kits (148) or TRIzol (149). Upon isolation, miRNAs can be stored at –70°C and remain stable for up to 1 year (148). There are several platforms available for miRNA profiling, including microarray hybridization, qPCR, and next-generation sequencing (150). Microarray and qPCR are the most frequently used methodologies to investigate the expression of known miRNAs (151). Both methods have the advantages of being simple to use, relatively quick from RNA labeling to data generation, and relatively cost-effective (152). However, they rely on the availability and accurate annotation of miRNA sequences in databases for probe and primer design (150). Although more expensive, next-generation sequencing allows for the simultaneous detection of both known and novel miRNA species and offers high sensitivity (153). Furthermore, the single-nucleotide resolution of next-generation sequencing enables the identification of isomiRs, which are mature miRNA isoforms that differ from canonical ones in length, sequence, or both (154, 155), which change the targeting specificity of the miRNA (156).

Diverse studies have investigated the potential of circulating miRNAs as biomarkers for pediatric kidney diseases (157–160). For instance, one study identified 14 miRNAs that were significantly upregulated in the serum of patients with Wilms tumor. Interestingly, a signature based on *miR-100-5p* and *miR-130-3p* expression could differentiate these patients from healthy controls with accuracy, sensitivity, and specificity (159). Although the findings from this study and many other studies have provided compelling motivation to explore the potential of circulating miRNAs as biomarkers, several hurdles in the field need to be overcome before widespread clinical application. First, there is a relative lack of consensus between studies likely due to the absence of standardized methodology for purification (161) and analysis of samples (e.g., differences in miRNA profiling platforms, refs. 162, 163; or differences in smRNA-Seq library preparation methods, ref. 164). Second is the lack of large-sample-size studies and detailed investigations on specific diseases. Another important aspect is that the influence of confounding variables such as age, sex, and external factors (e.g., tobacco, alcohol, etc.) on miRNA profiles has not been fully explored (for an in-depth review, please refer to ref. 165).

On the therapeutic side, several miRNA-based drugs are currently in clinical trials but have not been granted FDA approval yet (166, 167). The main approaches for miRNA therapy involve restoration of miRNA levels using miRNA mimics, or inhibition of specific miRNAs using antagomiRs (168). One of the challenges associated with the development of miRNA-based therapeutics is the identification of miRNA candidates for each disease. Because multiple miRNAs are dysregulated in each disease, a careful analysis of patient samples in combination with in vitro and in vivo assays that address the pathophysiological mechanisms affected by the miRNAs in question should be performed for narrowing down the candidate miRNAs for therapeutic intervention (168, 169). Another challenge involves the development of strategies to improve in vivo stability and site-specific drug delivery with minimal toxicity and off-target effects. RNA molecules are chemically unstable due to the presence of the 2′-hydroxyl group on the pentose ring. To provide higher stability and protection from nucleases present in serum or the endocytic compartment of cells, biotech companies have generated RNA molecules with chemical modifications (2′-O-methyl group, phosphorothioate, or locked nucleic acids) in their backbone (166). As for in vivo delivery, technologies include lipid based (e.g., lipid nanoparticle and neutral liposome) and dendrimers conjugated to a targeting moiety, among many other strategies. Major challenges associated with miRNA delivery systems are immunotoxicity and target-specific affinity toward a disease site (170). Delivery strategies by various methods of administration (intraperitoneal, intravenous, and subcutaneous injections) or by using vectors containing kidney-specific and inducible promoters have been successfully used for selective kidney targeting and to avoid potential adverse effects in other tissues and organs (171, 172).

## Summary

There has been an explosion of information regarding miRNA biogenesis, the regulation of miRNA expression, and miRNA function since the initial discovery of miRNAs in 1993 (6, 7). This has been accompanied by an ever-increasing understanding of how miRNAs function both in normal physiology and in the pathophysiology of many diseases. It has become clear that dysregulation of miRNA expression disrupts early kidney development and is implicated in the pathogenesis of developmental kidney diseases, such as CAKUT and Wilms tumor. With recent developments in the use of miRNAs as biomarkers and as novel drug targets, insights into how miRNAs regulate kidney development and disease are critical to understanding how they might be utilized in novel diagnostic and therapeutic approaches to these diseases. To fully realize these efforts, future studies identifying the function of specific miRNAs in kidney development are critical, in addition to technologies to optimize targeting small oligonucleotide therapeutics to the kidney.

## Figures and Tables

**Table 1:**
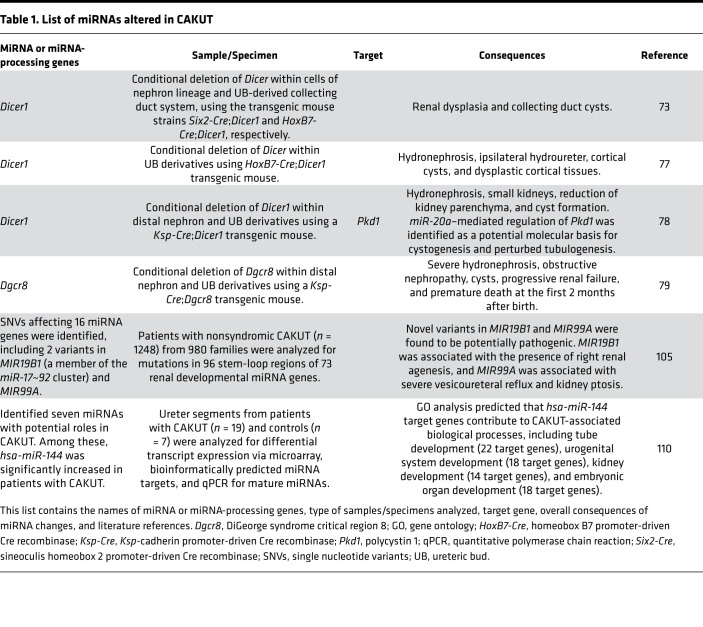
List of miRNAs altered in CAKUT

**Table 2:**
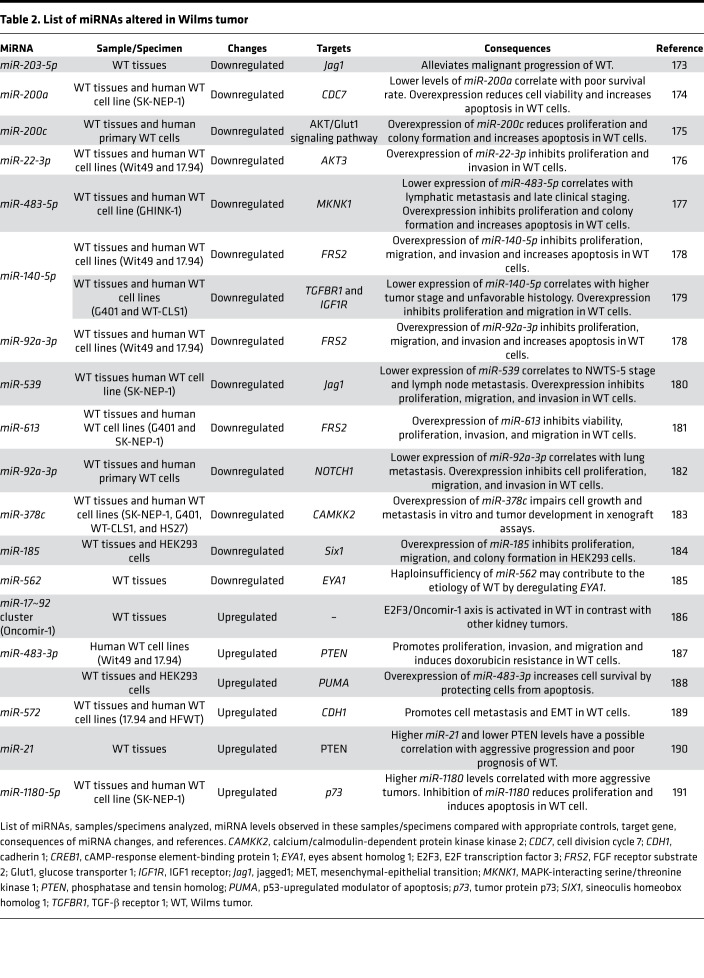
List of miRNAs altered in Wilms tumor

## Version 1 05/09/2022

Electronic publication
